# From statistics to clinics: the visual feedback of PROMIS® CATs

**DOI:** 10.1186/s41687-021-00324-y

**Published:** 2021-07-10

**Authors:** Maud M. van Muilekom, Michiel A. J. Luijten, Hedy A. van Oers, Caroline B. Terwee, Raphaële R. L. van Litsenburg, Leo D. Roorda, Martha A. Grootenhuis, Lotte Haverman

**Affiliations:** 1 Child and Adolescent Psychiatry & Psychosocial Care, Amsterdam Reproduction and Development, Amsterdam Public Health, Emma Children’s Hospital, Amsterdam UMC, University of Amsterdam, Postbox 22660, 1100 DD Amsterdam, The Netherlands; 2 Epidemiology and Data Science, Amsterdam UMC, Vrije Universiteit, Amsterdam, The Netherlands; 3 Princess Máxima Center for Pediatric Oncology, Utrecht, The Netherlands; 4 Pediatric Oncology, Cancer Center Amsterdam, Emma Children’s Hospital, Amsterdam UMC, Vrije Universiteit, Amsterdam, The Netherlands; 5 Amsterdam Rehabilitation Research Center | Reade, Amsterdam, The Netherlands

**Keywords:** Visual feedback, Patient reported outcome measures (PROMs), Patient-reported outcomes measurement information system (PROMIS®), Computerized adaptive testing (CAT), Clinicians, Pediatric patients and parents

## Abstract

**Background:**

To reduce the burden of completing Patient-Reported Outcome Measures (PROMs), PROMIS® Computerized Adaptive Tests (CATs) are being implemented in pediatric clinical practice. We aimed to develop recommendations for visual feedback options for PROMIS CATs on individual item and domain score level as an evidence-based feedback recommendation for PROMIS CATs is lacking.

**Methods:**

Focus groups were held with clinicians who use the KLIK PROM portal. Literature-based feedback options were provided to initiate group discussion. Data was analyzed using thematic coding method. Additionally, a questionnaire was sent out to assess patients’ (12-18y) and parents’ (child 0-18y) preference for individual item feedback. Data was analyzed using descriptive statistics.

**Results:**

Six focus groups were held (*N* = 28 clinicians). Regarding individual item feedback, showing the complete item bank, with only responses to administered items in traffic light colors was preferred. For domain scores, line graphs were preferred, including numerical (T-)scores, reference and cut-off lines, and traffic light colors. Separate graphs per domain, ranked in order of importance and harmonization of directionality (‘higher = better’) were considered important. Questionnaire results (*N* = 31 patients/*N* = 131 parents) showed that viewing their own item responses was preferred above receiving no item feedback by 58.1% of the patients and 77.1% of the parents.

**Conclusions:**

Based on the outcomes and after discussion with the Dutch-Flemish PROMIS National Center, recommendations for PROMIS CAT feedback options were developed. PROMIS CATs can now be used in clinical practice to help clinicians monitor patient outcomes, while reducing the burden of completing PROMs for patients significantly.

## Background

With the systematic use of Patient Reported Outcome Measures (PROMs, questionnaires measuring the patients’ view of their health status) in the consultation room, symptoms, physical and psychosocial functioning of patients can be monitored and discussed. When necessary, interventions can subsequently be offered timely [1, 2]. The use of PROMs in clinical practice has been shown beneficial as it resulted in increased discussion of patient outcomes and enhanced patient-clinician communication [3, 4], higher patient satisfaction [2], better Health Related Quality of Life (HRQOL) [5], and improved treatment outcomes including survival [5, 6].

Even though PROMs are increasingly used in clinical practice, several challenges with PROMs have been identified (van Muilekom, M. M., Teela, L., van Oers, H. A., Grootenhuis, M. A., & Haverman, L: Patients’ and parents’ perspective on the implemented KLIK PROM portal in clinical practice, unpublished) [7–9]. For example, available PROMs are often considered burdensome due to questionnaire length and irrelevancy and repetitiveness of questions. Additionally, when patients have multiple chronic conditions and thus have to complete PROMs for multiple diseases, scores of these PROMs cannot be compared due to different scoring methods.

To overcome these challenges and harmonize all existing PROMs into one assessment system, the Patient-Reported Outcomes Measurement Information System (PROMIS®) was developed by a consortium of US research centers, together with the National Institute of Health [10, 11]. PROMIS is a generic measurement system, consisting of various item banks, for adults and children, that measure separate domains representing physical, mental and social health (e.g., depression, pain interference) [12]. The item banks are based on Item Response Theory (IRT) modelling, where items are ordered by their difficulty and discriminative ability and scaled onto a single metric, which enables Computerized Adaptive Testing (CAT). With CAT, items are presented to patients based on responses to previously administered items. The computer estimates the domain score after each item, and when this score reaches a pre-defined precision, the CAT stops. Hence, patients only need to answer a small number of items (usually 5–7) per PROMIS item bank to get an accurate and reliable T-score [13]. Responses to remaining, non-administered items can be predicted (predicted responses) using the IRT model.

**Fig. 1:**
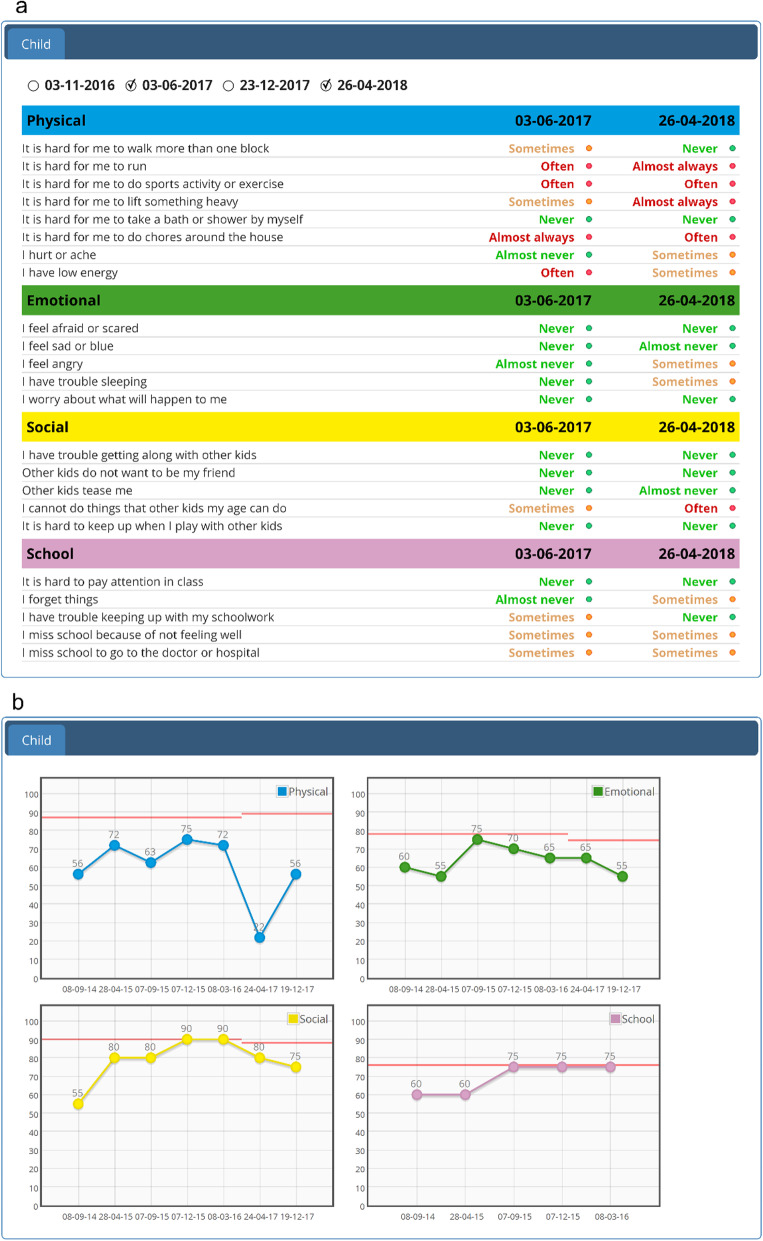
Current PROM individual item (**1a**) and domain score (**1b**) feedback in the KLIK ePROfile

To facilitate the use of the PROMIS item banks in clinical practice in the Netherlands, a large number of PROMIS item banks were translated into Dutch-Flemish and validated [8, 14–16]. In 2019, the Dutch-Flemish pediatric PROMIS item banks were implemented in the Netherlands through the KLIK PROM portal [17–19], after linking KLIK to the Dutch-Flemish Assessment Center to enable CAT [20]. KLIK is an online portal (www.hetklikt.nu or www.klik-uk.org) where patients and/or caregivers complete PROMs regarding symptoms, HRQOL, physical and psychosocial functioning. Responses are visualized in the KLIK ePROfile, on individual item level (with traffic light colors: green – no problems, orange – some problems, red – many problems) and domain score level (with graphs including a reference line) [20] (Fig. 1). It is essential that this visual feedback is easy-to-understand, as clinicians subsequently need to interpret the scores of different PROMs and discuss the feedback with the patients during consultation. However, for PROMIS CATs, new visual feedback options for the KLIK ePROfile are required, as an evidence-based feedback recommendation for PROMIS CATs is lacking.

Until now, several studies have investigated the visual feedback of PROMs in general, and current knowledge has been summarized [21, 22]. Two studies in an adult oncology and rheumatology setting showed that individual item feedback immediately attracts clinicians’ attention to specific problems, especially when using colors [23, 24]. Regarding domain score feedback, line graphs were most preferred to show change over time [25–31]. However, bar charts, tables or textual reports might be good alternatives [30, 32–34]. Meaningful descriptive labeling of axes, harmonization of directionality (higher is better: upward trend indicates better functioning), highlighting deviating results with colors and inclusion of a reference population were all identified as important aspects of visual feedback [27–30, 35, 36]. Concerning feedback of PROMIS specifically, some studies have described how they visualized PROMIS domain score (T-score) feedback when using PROMIS item banks in adult orthopedic, oncology, cardiac and gastrointestinal clinical practice [21, 37–40], where line graphs including reference to a norm population [21], textual reports of T-scores [21, 38], symptom cards [39] and heat maps [37, 40] were used. Showing T-scores in order of importance, with the most deviating T-score first, was described to be helpful in two studies [39, 40]. Only one study in adult orthopedic care provided individual item feedback of PROMIS CATs to patients and clinicians, but they did not explore preferences of their participants regarding this feedback [37].

To conclude, several studies have investigated feedback of PROMs in general and some described how they provide feedback when using PROMIS item banks in clinical practice. However, to our knowledge no studies were performed that explored preferences for PROMIS CAT feedback specifically. Thus, more insight is needed into optimal PROMIS CAT feedback and, therefore, this study aimed to develop recommendations for individual item and domain score feedback for PROMIS CATs.

## Methods

### Design

A mixed method design was used by combining qualitative and quantitative methodologies in two steps: 1) Focus groups with clinicians and 2) a questionnaire for pediatric patients and their parents. This study was approved by the Medical Ethics Committee of the Amsterdam University Medical Centers (Amsterdam UMC), location AMC. Informed consent was obtained from all participating clinicians and patients/parents.

### Participants and procedure

#### Focus groups

Participants were recruited between September and November 2018 using a purposive sampling method. The aim was to include clinicians from diverse disciplines (e.g., physicians, psychologists, social workers) who use KLIK in the Emma Children’s Hospital Amsterdam UMC or Princess Máxima Center. An invitation e-mail (explaining the goal of the study and including optional data for focus groups) was sent to all clinicians and a reminder e-mail was sent to clinicians who had not responded after 3 weeks. Interested clinicians could reply to the email and sign up to participate. Thereafter, clinicians were allocated to one of the focus groups, where ideally three to six participants [41] and a mix of different disciplines was pursued. All applicants from the Princess Máxima Center were admitted to one focus group during their standard multidisciplinary meeting in their own center due to limited time.

Focus groups consisted of a group discussion guided by two moderators (MMvM and MAJL) using a topic guide in PowerPoint. Both moderators were trained in performing focus groups. First, a short recapitulation of KLIK and the current PROM feedback options was provided. Thereafter, PROMIS and the principles of CAT were explained, enabling clinicians to understand why new feedback was necessary. To obtain clinicians’ input on PROMIS CAT feedback, four options for individual item and five options for domain score feedback were shown, based on or adapted from previous studies [39, 42–44]. Questions were provided to clinicians (‘What appeals to you in this option?’, ‘What do you miss in this option?’) to initiate the discussion about the feedback options (e.g., including predicted responses for non-administered items and providing reference lines). Furthermore, clinicians were asked to describe their optimal feedback option. The duration of each focus group was approximately 90 min. All focus groups were audio recorded.

#### Questionnaire

**Fig. 2:**
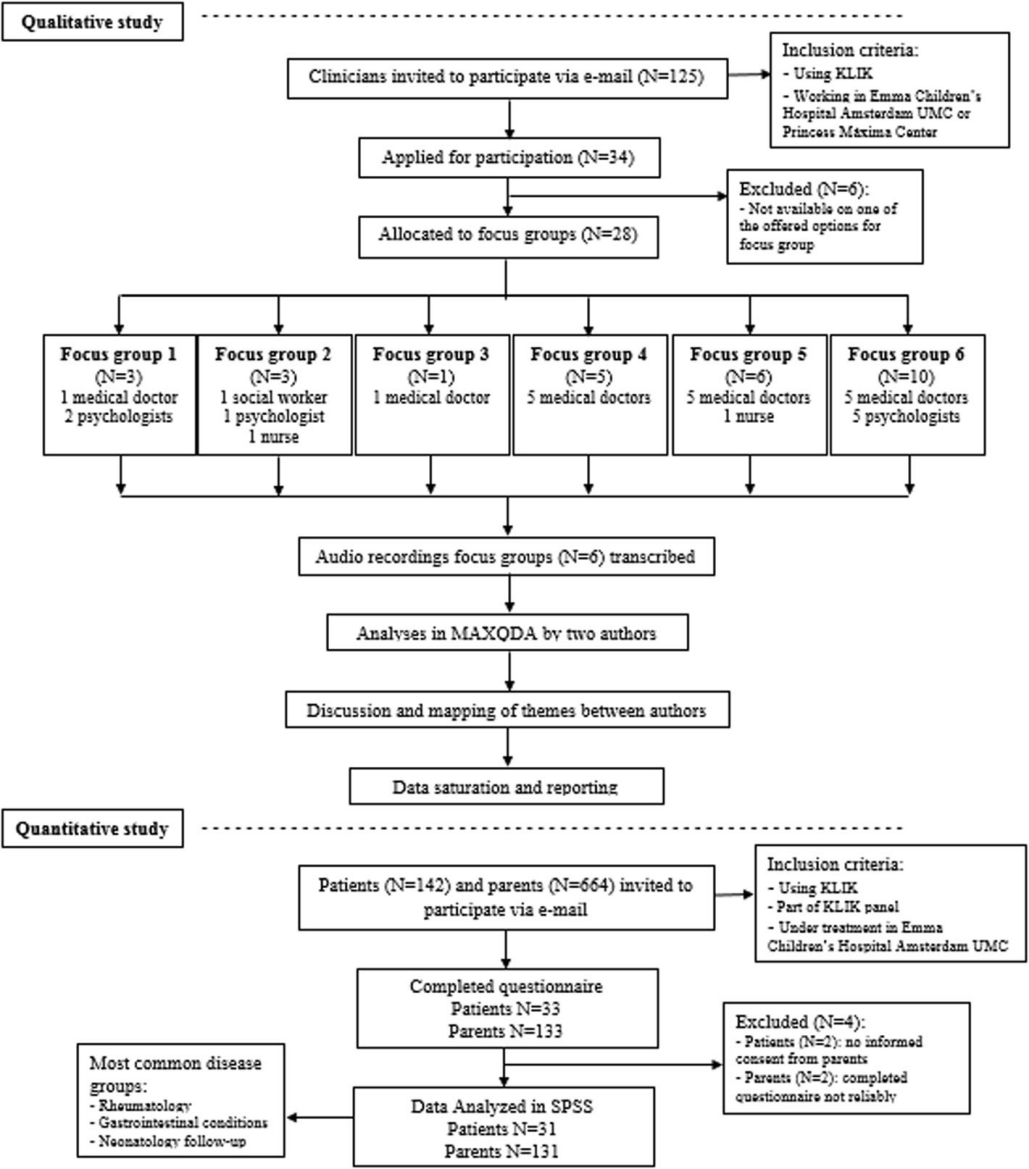
Study and participant flow chart of the qualitative (focus groups) and quantitative (questionnaire) study

To receive patients’ and parents’ opinion on PROMIS CAT feedback, an online questionnaire was sent out between June and December 2019. All patients (12–18 years) and parents (of children 0–18 years) who use KLIK as standard part of care in the Emma Children’s Hospital Amsterdam UMC, completed KLIK PROMs at least once, and were part of the KLIK panel (during registration on the KLIK PROM portal they could indicate they were willing to participate in research projects) were invited by email (Fig. 2). Participants completed the questionnaire anonymously. Three reminder emails were sent over the course of 6 months to patients and parents who had not yet completed the questionnaire. All patients and parents provided informed consent and received a gift card after participation.

**Fig. 3:**
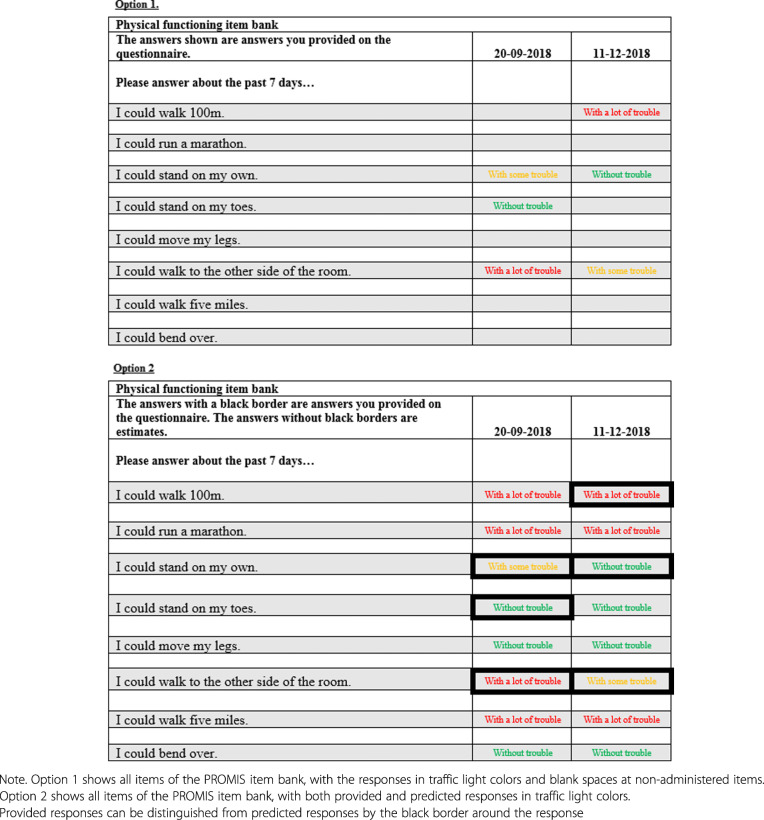
Two options of individual item feedback shown to patients and parents in questionnaire

The questionnaire (separate versions for patients and parents) was developed as part of a larger study that aimed to assess KLIK users’ opinion about several aspects of the KLIK PROM portal. Three questions were included in this study that focused on the feedback of PROMIS CATs. Only questions about individual item feedback could be asked, as patients and parents currently do not receive domain score feedback in their KLIK ePROfile. A short explanatory text about the working mechanism of PROMIS CATs was provided after which the following three questions were asked: 1) ‘Would you like to see your responses in the KLIK ePROfile?’ (yes/no), 2) ‘Would you like to see all items of the item bank in the KLIK ePROfile?’ (yes/no), and 3) ‘Which of the two figures provided would you like to see in the KLIK ePROfile?’ (option 1/option 2, Fig. 3). For every question there was the possibility to add an explanation or provide additional remarks.

### Analyses

#### Focus groups

Audio recordings were transcribed verbatim (and data was anonymized) and two authors independently read and analyzed the transcripts with the qualitative data analysis tool MAXQDA (2018) using a thematic coding method [45]. Analyses included the following steps; 1) marking parts of the transcript related to the subject matter 2) generating initial codes to organize data into meaningful groups, 3) searching for themes and collating codes into the identified themes, 4) reviewing and refining themes into main themes and subthemes, 5) defining the final themes.

After analyzing all transcripts independently, analyses were discussed between two authors until consensus on the themes was reached. Data saturation was considered attained when, during analyses of the planned focus groups, no new themes emerged. If new themes did emerge, new focus groups would be planned until data saturation was reached.

#### Questionnaire

Descriptive analyses (percentages yes/no or option 1/option 2) were performed on the three questions to gain insight in participants’ preference for feedback of PROMIS CATs by using the Statistical Package for Social Sciences (SPSS) version 25.0.

### Development of recommended feedback options for PROMIS CATs

After analyzing the results of the focus groups and the questionnaire, a preliminary recommended individual item and domain score feedback option was developed. Thereafter, these feedback options were discussed with the Dutch-Flemish PROMIS National Center and an expert on data visualization was consulted, to develop a final recommendation for feeding back PROMIS CATs on individual item and domain score level.

## Results

### Focus groups

In the upper part of Fig. 2 the study and participant flow chart of the focus groups is shown. In total, 28 clinicians participated in six focus groups (response rate: 22.4%). Characteristics of clinicians are shown in Table 1. On average, clinicians used KLIK for 5.2 years (range: 0.3–7.4). The majority of clinicians worked in the Emma Children’s Hospital (64.3%) and most clinicians were employed as medical doctor (60.7%) or psychologist (28.6%). Data saturation was attained as no new themes emerged after analyzing the final planned focus group. Table 2 shows the most important themes and corresponding examples of statements expressed by clinicians about individual item and domain score feedback of PROMIS CATs.

**Table 1:** Characteristics of participating clinicians in six focus groups

	Participants (***N*** = 28)	
	M	Range
**KLIK user since (years)**	5.2	0.3–7.4
	**N (%)**	
**Hospital**		
Emma Children’s Hospital	18 (64.3)	
Princess Máxima Center^a^	10 (35.7)	
**Discipline**		
Medical doctor	17 (60.7)	
Psychologist	8 (28.6)	
Nurse	2 (7.1)	
Social worker	1 (3.6)	

*Note*. ^a^Only 1 focus group (Focus group 6) was held in this hospital

**Table 2:** Themes and examples found in focus groups

Feedback	Themes	Focus group number	Examples
**Individual item**	All items	3	*“I would like to see all items in the feedback, as then there is the possibility to discuss also not completed items.”*
		1	*“For non-experienced clinicians who do not know the questionnaires it is nice to be able to see all items.”*
		6	*“Seeing all the items of the questionnaire provides the opportunity to use them as a conversation tool.”*
	Completed items	2	*“Only the responses to the items that the patient has completed should be fed back.”*
		5	*“Feedback of the responses to the completed items provides the opportunity to start a conversation with the patient.”*
	Colors	4	*“The use of traffic light colors helps me in focusing quickly on what is important.”*
		6	*“Seeing the traffic light colors is essential as it makes interpreting easy and simple.”*
	Predicted responses	1	*“The predicted responses provide too much information. If predicted responses are shown I would still want to check them and adjust them if needed, which would cost me more time!”*
		2	*“Feeding back predicted responses is very confusing for use in clinical practice, especially to discuss them with the patient. Perhaps in research predicted responses might be useful.”*
**Domain score**	Dots or lines	5	*“I think viewing lines between the dots that represent the domain scores for that time point is clearer and interpretation is easier.”*
	Numerical information	4	*“If the numerical domain (T-)scores are provided in the graph, this is very useful. Especially, as you can also use these scores in the report about the patient in the electronic health record.”*
	Reference line (and cut-offs)	3	*“A norm line makes the graph more insightful and clear.”*
		1	*“It is relevant to see the cut-off lines as well, as with these lines you can judge if a patient has a subclinical or clinical score.”*
	Colors	3	*“The use of traffic light colors makes the graph easier interpretable and provides a quick overview of how the patient is functioning.”*
		6	*“When the domain scores or cut-off lines are shown in traffic light colors you can see how good or bad the score of the patient is.”*
		2	*“Another option is to show the background of the graph in traffic light colors, in accordance with the cut-off lines, whilst showing the domain scores in a neutral color. In this way I can quickly see on what level the patient is functioning.”*
	Combined or separate graphs	1	*“Separate graphs per domain are better, as the domains are so different from each other. Putting them together in one graph would result in oversimplification of the findings.”*
		4	*“It is more difficult to discuss the outcomes if they are all put in one graph.”*
	Order of importance	2	*“It would be very helpful if the graph where the most deviating domain score in the clinical direction is found and thus needs most attention, is ranked in order of importance and is shown first.”*
	Directionality	4	*“For me it is important that if several graphs are shown on one page that all lines are going in the same direction.”*
		5	*“I would prefer to see norm lines go up when functioning is better and go down when functioning is worse. In other words, higher is better.”*

#### Individual item feedback

In all focus groups clinicians indicated that feedback of individual items is essential for the use of PROMs in clinical practice. Clinicians use the items to start a dialogue (as a conversation tool), to understand the domain scores that are provided and to discuss specific problems. Therefore, it was important for them to obtain individual item feedback for PROMIS CATs.

Even though with PROMIS CATs not all items are administered to patients, it was important for clinicians to have the possibility to see *all items* of the item bank in the feedback. According to the clinicians, the responses to the *completed items* in the CAT should be fed back, preferably in traffic light *colors*, where items on which no problems are reported are shown in green, items on which some problems are reported are shown in orange and items on which many problems are reported are shown in red. In this way clinicians can quickly see if the patient has problems with certain symptoms or aspects of their daily functioning. The option to include *predicted responses* in the feedback (which is possible using the IRT model) for non-administered items, was unanimously rejected. Reasons were that predicted responses are not recognizable for patients and can be confronting and confusing. A suggestion provided by clinicians was to leave blank spaces at items that were not administered. Over time, clinicians can then easily see which items were administered with every CAT completion.

#### Domain score feedback

Regarding the domain score feedback of PROMIS CATs it became clear, by discussing the several options provided, that clinicians had a preference for graphical over textual options. Graphs were seen as clearer and easier to interpret, and the option to show domain scores longitudinally in one graph was desired.

A large majority of clinicians preferred to see the domain scores over time in graphs as *dots* connected with *lines,* though for a few clinicians this connection did not matter, as long as there was a graphical feedback option. According to the clinicians, the *numerical domain (T-)score* should be shown with each dot, as these scores help improve the interpretation of scores and can be easily included in the health record. In addition to the individual patient’s domain scores, inclusion of a *reference line* was valued by all clinicians, in order to make a comparison with a reference group. To be able to judge the severity of scores deviating from the reference line, several options were discussed, for example showing the dots in traffic light *colors* or adding *cut-off lines* in traffic light *colors* indicating subclinical (moderate deviation from norm) or clinical (severe deviation from norm) scores. An additional suggestion provided by clinicians, was to give areas in the background of the graph traffic light *colors* (similar to a heat map), in accordance with the cut-off lines, and the dots of the domain scores in neutral colors. Although at first participating psychologists thought that the use of colors was confronting for patients, they later agreed that it is useful to quickly assess if a patient deviates from the reference group.

As PROMIS measures domains on similar scales (T-score metric), it is possible to display multiple domain scores in one graph. However, this was considered unclear and difficult to interpret. Clinicians preferred *separate graphs* per domain, all shown on one page, and if possible ranked in *order of importance*. They indicated that graphs where the most deviating scores were found on a domain should be presented first, by which clinicians can easily see which domains need most attention. The last topic that came up was the *directionality* of lines. A large majority of clinicians indicated that it is important for them to harmonize the directionality in all graphs. They preferred to see lines where an upwards trend represents an improvement in functioning (higher is better). To do this, they suggested to reverse the scale on the y-axis for some domains (e.g., for anxiety, where higher scores indicate higher anxiety levels).

### Questionnaire

In the lower part of Fig. 2 the study and participant flow chart of the questionnaire is presented. In total, completed questionnaires of 31 patients (response rate: 21.8%) and 131 parents (response rate: 19.7%) were analyzed. Since participants completed the questionnaire anonymously, no information on sociodemographic characteristics of participants was available, nor information about the non-participants. Table 3 shows the results of the questionnaire for both patients and parents.

**Table 3:** Questionnaire results for patients and parents

	Patients			Parents		
Question	***N***	Yes (%)	No (%)	***N***	Yes (%)	No (%)
Would you like to see your responses?	31	18 (58.1)	13 (41.9)	131	101 (77.1)	30 (22.9)
Would you like to see all items of the item bank?	31	13 (41.9)	18 (58.1)	131	55 (42.0)	76 (58.0)
		**Option 1 (%)**	**Option 2 (%)**		**Option 1 (%)**	**Option 2 (%)**
Which of the two figures provided would you like to see?	31	16 (51.6)	15 (48.4)	129^a^	96 (74.4)	33 (25.6)

*Note*. ^a^ Two parents were excluded as they indicated in the explanation box that they did not understand the figures at all

#### Patients (12–18 years)

The majority of patients (58.1%) indicated they would like to see their item responses fed back in the KLIK ePROfile, as they provide clarity and insight into their functioning. Less than half of the patients (41.9%) would like to see all items of the item bank. In their opinion the not completed items were unnecessary to show. Finally, 51.6% of the patients preferred not to see predicted responses (option 1). As an explanation for this choice, patients mentioned that option 2 was very unclear and contained too many details.

#### Parents

Most parents (77.1%) preferred to see their responses to the items. An explanation for this preference was that these provide insight into the functioning of their child, especially when the responses of several measurement occasions are shown. In accordance with patients, less than half of the parents (42.0%) would like to see all items of the item bank, as they think that viewing the not completed items is not of added value. The majority of parents (74.4%) preferred not to see predicted responses (option 1). Explanations were that option 2 was too complicated to read and contained too many details which makes the option unclear.

### Recommended feedback options for PROMIS CATs

**Fig. 4:**
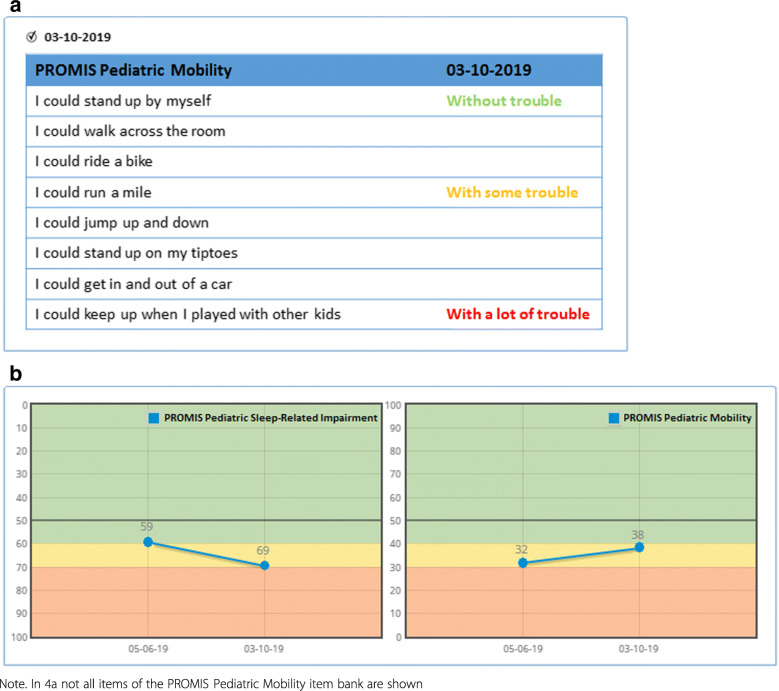
Preliminary recommended individual item (**4a**) and domain score (**4b**) feedback of PROMIS CATs

Based on the outcomes of the focus groups and questionnaire a preliminary recommended individual item and domain score feedback option was developed (Fig. 4). Regarding *individual item feedback*, all items of the item bank are shown (based on the preference of clinicians), with the responses of patients shown in traffic light colors and blank spaces at items that were not administered. Regarding *domain score feedback* separate graphs per domain are shown on one page. The graphs include dots (with numerical domain (T-)scores) connected by a blue line with a background in traffic light colors (heat map), showing the deviation of the reference line in orange (moderate) and red (severe). In addition, on the y-axis the scales are reversely presented for some domains in order to harmonize the directionality of the lines in all graphs (higher is less symptoms or better functioning).

**Fig. 5:**
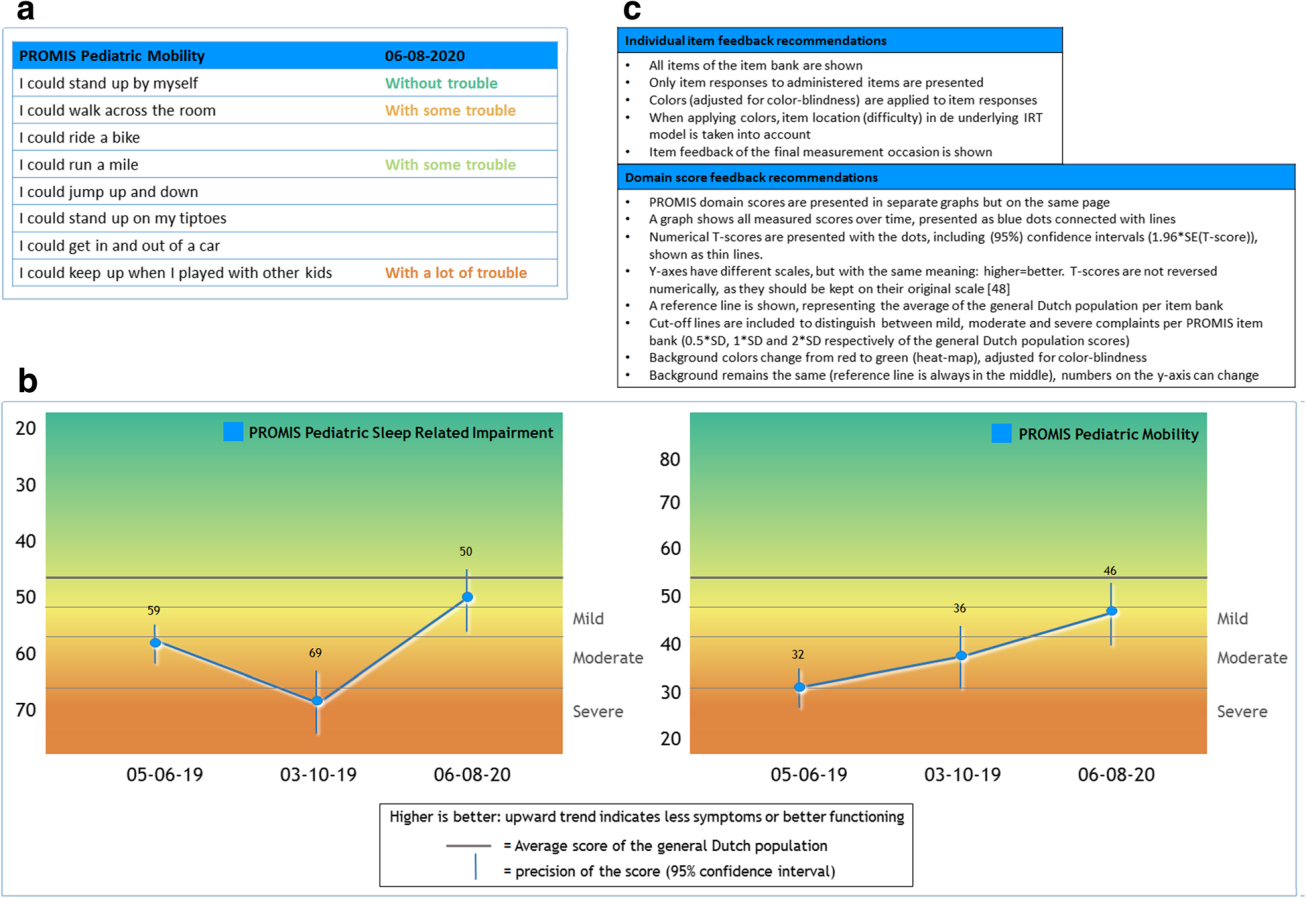
Final recommended individual item (**5a**) and domain score (**5b**) feedback of PROMIS CATs including recommendation boxes (**5c**) [47]

After discussion with the Dutch-Flemish PROMIS National Center and consultation of a data visualization expert, some adjustments were made and a final recommendation was developed (Fig. 5). The most important adaptation is that a wider color-palette is used, which was adjusted for people with color-blindness [46]. Additionally, for individual item feedback, colors are now applied to the items based on the item location (difficulty) in the underlying IRT model. For domain score feedback, 95% confidence error bars (included with the scores), an extra cut-off line and y-axis labels (mild, moderate, severe, based on deviation of the reference line) were added.

## Discussion

To facilitate the use of PROMIS item banks in clinical practice, this study developed preliminary recommendations to feed back PROMIS CATs, on individual item and domain score level. Regarding individual item feedback, results displayed clinicians’ preference for showing all items of the item bank. Both clinicians and patients/parents agreed that only responses to administered items (in traffic light colors) and no predicted responses for non-administered items should be shown. Graphs were preferred for domain score feedback, which should include dots connected by lines, numerical domain (T-)scores, and a reference line. Deviating scores should be distinguishable by the use of cut-off lines, dots or the background of the graph in traffic light colors. Separate graphs per domain, ranked in order of importance and harmonization of directionality (‘higher is better’) were also preferred. To our knowledge, this was the first study that developed feedback options of PROMIS CATs, which is an important step for implementation in clinical practice. Based on the results and after discussion with the Dutch-Flemish PROMIS National Center, a final recommended individual item and domain score feedback option for PROMIS CATs were developed, which was also implemented in the KLIK PROM portal. In this final version color-blindness was taken into account for both individual item and domain score feedback.

Individual item feedback was regarded as essential by clinicians to discuss PROMs in clinical practice as items can be used as a conversation tool and immediately attract clinicians’ attention to problems, especially when using traffic light colors. Patients and parents also preferred to see their responses on individual items in the KLIK ePROfile. This finding is in accordance with previous studies on feeding back individual items of PROMs in clinical practice [23, 24, 37].

There are two challenges regarding feeding back individual items of PROMIS CATs. First, clinicians indicated that they preferred the option to see all items of the item bank, where both administered and non-administered items are shown. Even though this option was appropriate for pediatric item banks (maximum of 34 items per item bank), which was the focus of this study, a challenge arises when individual items of adult PROMIS item banks are to be fed back, as these item banks sometimes consist of more than hundred items. A solution could be to only present the responses in traffic light colors of the items that have been administered over time, and not all items of the item bank. This might also be an option for patients and parents, as they indicated they do not necessarily want to see all items of the item bank. For them there is the possibility in for example the KLIK PROM portal to show an adjusted individual item feedback option. The second challenge is the use of traffic light colors for item responses. In the preliminary individual item feedback option developed in this study, colors were applied to item responses based on the response category (i.e. responses “without trouble” are always shown in green and responses “with a lot of trouble” are always shown in red). An alternative – implemented in the final recommendation – is to take the item location (difficulty) of the underlying IRT model into account. For example, the response “with a lot of trouble” of a very ‘difficult’ item (e.g., “I could run a mile”) may not necessarily indicate a problem and would not be presented in red.

Regarding domain score feedback, a strong preference of clinicians was found for graphs, as graphs can easily display longitudinal data, which was reported in previous studies as well [21, 25–31]. All other options like tables, textual reports or symptom cards were immediately discarded, which is in contrast with earlier research [21, 30, 32–34, 37–40]. Reasons reported in the focus groups were that these alternatives take more time to interpret, are more difficult to discuss with the patient and cannot present more than two measurement occasions without losing overview. All other features that were reported as important, e.g., reference to a norm group, highlighting deviating results, harmonization of directionality and ranking graphs in order of importance, are in accordance with previous literature on PROM feedback [21, 27–30, 35, 36].

There are three challenges regarding domain score feedback. First, clinicians indicated they preferred to see a reference line in the graph, including cut-off points to judge the severity of deviation. However, which reference line and cut-off values should be fed back is a point of discussion. They can be based on the US metric (reference score of 50 and SD of 10) or based on the average scores of the country-specific general population (reference score and SD differ a little bit per item bank). Additionally, how many cut-off points should be shown? And what labels should be included? In the final recommended domain score feedback option we chose to include the average and SD of the general Dutch population. Three cut-off points with labels ‘mild’, ‘moderate’ and ‘severe’ were chosen (based on 0.5*SD, 1*SD, and 2*SD) in accordance with the suggested score interpretations on the Healthmeasures website (www.healthmeasures.net). However, these cut-offs could be adapted once, for example, bookmarking study results (cut-offs based on patient input) are available. Second, for the ranking of graphs in order of importance, it should be further explored whether ranking should be based on deviation of scores from the reference group, on relevance of the domain for the patient or clinician, or based on recent changes in scores. Third, no consensus was reached in the focus groups on how to indicate deviating scores (either dots, cut-off lines or background in traffic light colors). The background coloring (heat map) with cut-off lines was chosen as final recommendation, as this is easiest to comprehend and takes least time to interpret. This point however, needs to be discussed and evaluated again in the future.

There are some limitations to this study that should be mentioned. First, the sample could be biased as both clinicians and patients/parents were KLIK users and they were thus already used to the feedback that is currently provided for other PROMs. This might have influenced their opinion about their optimal feedback option, which is visible in the similarities between the recommended feedback options and the feedback options used in KLIK. However, as the findings are in accordance with previous literature and as clinicians also came up with new important features that are currently not available in KLIK, it can be assumed that the developed feedback option represents the opinion of a wider audience. Second, the clinician sample was somewhat skewed and consisted mainly of medical doctors. However, this is representative of the disciplines that use KLIK in clinical practice, where medical doctors are also the main user group. Only nurses were relatively underrepresented. Third, response rates for the questionnaire were low and only a small number of pediatric patients participated. Even though reminder emails were sent, future studies could consider to approach patients by telephone or emphasize the importance of their participation more to increase the response rates. Fourth, patients’ and parents’ perspectives were not optimally taken into account by using a questionnaire only. For example, it was difficult to explain the working mechanism of PROMIS CATs to patients and parents in a questionnaire and to verify their understanding of the concept. Especially from the responses of patients in the explanation boxes this lack of understanding was noticed, and this might explain the non-conclusive results regarding the questions about not answered items and predicted responses. In addition, they could only provide their opinion about individual item feedback, as domain score feedback is currently not shown to patients and parents in KLIK. Since several studies have shown patients’ and parents’ preference for viewing domain score feedback for other PROMs [21, 25, 28, 29, 31, 32], we decided to include domain score feedback (without reference lines) for patients and parents in KLIK in the near future. In future, preferably qualitative studies, the developed recommendations (especially the domain score feedback) should then be discussed with patients and parents as well.

## Conclusions

In conclusion, this study developed recommendations for feedback options for PROMIS CATs. Based on the preferences of clinicians and patients/parents and discussion with the Dutch-Flemish PROMIS National Center, an individual item and domain score feedback option were developed. In future studies, the current recommendations should be investigated with clinicians, patients and parents on interpretation accuracy and effectiveness in clinical practice. The availability of these feedback options facilitates using PROMIS CATs in clinical practice. With CAT, patients only have to complete a small number of items per domain that are applicable to their situation, which reduces the burden of completing PROMs significantly. For clinicians the developed simple and clear feedback of PROMIS CATs might help in monitoring and discussing patient outcomes, which contributes to optimal care for patients.

## Data Availability

The datasets used and/or analyzed during the current study are available from the corresponding author on reasonable request.

## Abbreviations

PROMs: Patient Reported Outcome Measures
HRQOL: Health Related Quality of Life
PROMIS®: Patient-Reported Outcomes Measurement Information System
IRT: Item Response Theory
CAT: Computerized Adaptive Testing
Amsterdam UMC: Amsterdam University Medical Centers
SPSS: Statistical Package for Social Sciences
SD: Standard Deviation

## References

[1] Reeve, B. B., Wyrwich, K. W., Wu, A. W., Velikova, G., Terwee, C. B., Snyder, C. F., … Butt, Z. (2013). ISOQOL recommends minimum standards for patient-reported outcome measures used in patient-centered outcomes and comparative effectiveness research. *Quality of Life Research*, *22*(8), 1889–1905. 10.1007/s11136-012-0344-y. 10.1007/s11136-012-0344-y 23288613

[2] Kotronoulas G, Kearney N, Maguire R, Harrow A, Di Domenico D, Croy S (2014). What is the value of the routine use of patient-reported outcome measures toward improvement of patient outcomes, processes of care, and health service outcomes in cancer care? A systematic review of controlled trials. Journal of Clinical Oncology.

[3] Marshall S, Haywood K, Fitzpatrick R (2006). Impact of patient-reported outcome measures on routine practice: a structured review. Journal of Evaluation in Clinical Practice.

[4] Valderas J, Kotzeva A, Espallargues M, Guyatt G, Ferrans C, Halyard M (2008). The impact of measuring patient-reported outcomes in clinical practice: a systematic review of the literature. Quality of Life Research.

[5] Basch, E., Deal, A. M., Kris, M. G., Scher, H. I., Hudis, C. A., Sabbatini, P., … Schrag, D. (2016). Symptom monitoring with patient-reported outcomes during routine cancer treatment: a randomized controlled trial. *Journal of Clinical Oncology*, *34*(6), 557–565. 10.1200/JCO.2015.63.0830. 10.1200/JCO.2015.63.0830 PMC4872028 26644527

[6] Basch E, Deal AM, Dueck AC, Scher HI, Kris MG, Hudis C, Schrag D (2017). Overall survival results of a trial assessing patient-reported outcomes for symptom monitoring during routine cancer treatment. JAMA.

[7] Teela, L., van Muilekom, M. M., Kooij, L. H., Gathier, A. W., van Goudoever, J. B., Grootenhuis, M. A., … van Oers, H. A. (2020). Clinicians’ perspective on the implemented KLIK PROM portal in clinical practice. *Quality of Life Research*. 10.1007/s11136-020-02522-5. 10.1007/s11136-020-02522-5 PMC8528749 32468402

[8] Terwee C, Roorda L, De Vet H, Dekker J, Westhovens R, Van Leeuwen J (2014). Dutch–Flemish translation of 17 item banks from the patient-reported outcomes measurement information system (PROMIS). Quality of Life Research.

[9] Gamper E-M, Martini C, Petersen MA, Virgolini I, Holzner B, Giesinger JM (2019). Do patients consider computer-adaptive measures more appropriate than static questionnaires?. Journal of Patient-Reported Outcomes.

[10] Cella D, Yount S, Rothrock N, Gershon R, Cook K, Reeve B, Ader D, Fries JF, Bruce B, Rose M, PROMIS Cooperative Group (2007). The patient-reported outcomes measurement information system (PROMIS): Progress of an NIH roadmap cooperative group during its first two years. Medical Care.

[11] Cella, D., Riley, W., Stone, A., Rothrock, N., Reeve, B., Yount, S., … PROMIS Cooperative Group (2010). The patient-reported outcomes measurement information system (PROMIS) developed and tested its first wave of adult self-reported health outcome item banks: 2005–2008. *Journal of Clinical Epidemiology*, *63*(11), 1179–1194. 10.1016/j.jclinepi.2010.04.011. 10.1016/j.jclinepi.2010.04.011 PMC2965562 20685078

[12] Riley WT, Rothrock N, Bruce B, Christodolou C, Cook K, Hahn EA, Cella D (2010). Patient-reported outcomes measurement information system (PROMIS) domain names and definitions revisions: further evaluation of content validity in IRT-derived item banks. Quality of Life Research.

[13] Cella D, Gershon R, Lai J-S, Choi S (2007). The future of outcomes measurement: item banking, tailored short-forms, and computerized adaptive assessment. Quality of Life Research.

[14] Haverman L, Grootenhuis MA, Raat H, van Rossum MA, van Dulmen-den Broeder E, Hoppenbrouwers K (2016). Dutch–Flemish translation of nine pediatric item banks from the patient-reported outcomes measurement information system (PROMIS)®. Quality of Life Research.

[15] Terwee C, Crins M, Boers M, de Vet H, Roorda L (2019). Validation of two PROMIS item banks for measuring social participation in the Dutch general population. Quality of Life Research.

[16] Luijten, M. A., Terwee, C. B., van Oers, H. A., Joosten, M. M., van den Berg, J. M., Schonenberg-Meinema, D., et al. (2019). Psychometric properties of the pediatric patient-reported outcomes measurement information system (PROMIS®) item banks in a Dutch clinical sample of children with juvenile idiopathic arthritis. *Arthritis Care and Research*, *72*(12), 1780–1789. 10.1002/acr.24094 10.1002/acr.24094 PMC7756261 31628731

[17] Engelen, V., Detmar, S., Koopman, H., Maurice-Stam, H., Caron, H., Hoogerbrugge, P., … Grootenhuis, M. (2012). Reporting health-related quality of life scores to physicians during routine follow-up visits of pediatric oncology patients: is it effective? *Pediatric Blood & Cancer*, *58*(5), 766–774. 10.1002/pbc.23158. 10.1002/pbc.23158 21584933

[18] Haverman L, van Rossum MA, van Veenendaal M, van den Berg JM, Dolman KM, Swart J (2013). Effectiveness of a web-based application to monitor health-related quality of life. Pediatrics.

[19] Haverman L, van Oers HA, Limperg PF, Hijmans CT, Schepers SA, Nicolaas S (2014). Implementation of electronic patient reported outcomes in pediatric daily clinical practice: The KLIK experience. Clinical Practice in Pediatric Psychology.

[20] Haverman L, van Oers HA, van Muilekom MM, Grootenhuis MA (2019). Options for the interpretation of and recommendations for acting on different PROMs in daily clinical practice using KLIK. Medical Care.

[21] Fischer KI, De Faoite D, Rose M (2020). Patient-reported outcomes feedback report for knee arthroplasty patients should present selective information in a simple design-findings of a qualitative study. Journal of Patient-Reported Outcomes.

[22] Snyder C, Wu A (2017). Users’ guide to integrating patient-reported outcomes in electronic health records.

[23] Gilbert A, Sebag-Montefiore D, Davidson S, Velikova G (2015). Use of patient-reported outcomes to measure symptoms and health related quality of life in the clinic. Gynecologic Oncology.

[24] Sokka T (2016). Go, go, GoTreatlT!. Clinical and Experimental Rheumatology.

[25] Bantug ET, Coles T, Smith KC, Snyder CF, Rouette J, Brundage MD (2016). Graphical displays of patient-reported outcomes (PRO) for use in clinical practice: What makes a pro picture worth a thousand words?. Patient Education and Counseling.

[26] Brundage MD, Smith KC, Little EA, Bantug ET, Snyder CF (2015). Communicating patient-reported outcome scores using graphic formats: results from a mixed-methods evaluation. Quality of Life Research.

[27] Smith KC, Brundage MD, Tolbert E, Little EA, Bantug ET, Snyder CF (2016). Engaging stakeholders to improve presentation of patient-reported outcomes data in clinical practice. Support Care Cancer.

[28] Snyder C, Smith K, Holzner B, Rivera YM, Bantug E, Brundage M (2019). Making a picture worth a thousand numbers: recommendations for graphically displaying patient-reported outcomes data. Quality of Life Research.

[29] Snyder CF, Smith KC, Bantug ET, Tolbert EE, Blackford AL, Brundage MD, the PRO Data Presentation Stakeholder Advisory Board (2017). What do these scores mean? Presenting patient-reported outcomes data to patients and clinicians to improve interpretability. Cancer.

[30] Wu AW, White SM, Blackford AL, Wolff AC, Carducci MA, Herman JM (2016). Improving an electronic system for measuring PROs in routine oncology practice. Journal of Cancer Survivorship.

[31] McNair AG, Brookes ST, Davis CR, Argyropoulos M, Blazeby JM (2010). Communicating the results of randomized clinical trials: do patients understand multidimensional patient-reported outcomes?. Journal of Clinical Oncology.

[32] Cronin RM, Conway D, Condon D, Jerome RN, Byrne DW, Harris PA (2018). Patient and healthcare provider views on a patient-reported outcomes portal. Journal of the American Medical Informatics Association.

[33] Izard J, Hartzler A, Avery DI, Shih C, Dalkin BL, Gore JL (2014). User-centered design of quality of life reports for clinical care of patients with prostate cancer. Surgery.

[34] Fritz F, Ständer S, Breil B, Riek M, Dugas M (2011). CIS-based registration of quality of life in a single source approach. BMC Medical Informatics and Decision Making.

[35] Barthel D, Fischer K, Nolte S, Otto C, Meyrose A-K, Reisinger S (2016). Implementation of the Kids-CAT in clinical settings: a newly developed computer-adaptive test to facilitate the assessment of patient-reported outcomes of children and adolescents in clinical practice in Germany. Quality of Life Research.

[36] Tolbert E, Brundage M, Bantug E, Blackford AL, Smith K, Snyder C, PRO Data Presentation Stakeholder Advisory Board (2018). Picture this: presenting longitudinal patient-reported outcome research study results to patients. Medical Decision Making.

[37] Rothrock, N. E., Bass, M., Blumenthal, A., Gershon, R. C., Hanson, B., Joeris, A., … Vrahas, M. S. (2019). AO patient outcomes center: design, implementation, and evaluation of a software application for the collection of patient-reported outcome measures in orthopedic outpatient clinics. *JMIR Formative Research*, *3*(2), e10880. 10.2196/10880. 10.2196/10880 PMC6484265 30977735

[38] Wagner, L. I., Schink, J., Bass, M., Patel, S., Diaz, M. V., Rothrock, N., … Cella, D. (2015). Bringing PROMIS to practice: brief and precise symptom screening in ambulatory cancer care. *Cancer*, *121*(6), 927–934. 10.1002/cncr.29104. 10.1002/cncr.29104 PMC4352124 25376427

[39] Grossman LV, Mitchell EG (2017). Visualizing the patient-reported outcomes measurement information system (PROMIS) measures for clinicians and patients. AMIA annual symposium proceedings.

[40] Almario, C. V., Chey, W., Kaung, A., Whitman, C., Fuller, G., Reid, M., … Spiegel, B. M. R. (2015). Computer-generated versus physician-documented history of present illness (HPI): results of a blinded comparison. *The American Journal of Gastroenterology*, *110*(1), 170–179. 10.1038/ajg.2014.356. 10.1038/ajg.2014.356 PMC4289091 25461620

[41] Carlsen B, Glenton C (2011). What about N? A methodological study of sample-size reporting in focus group studies. BMC Medical Research Methodology.

[42] Rothrock, N. E., Amtmann, D., & Cook, K. F. (2020). Development and validation of an interpretive guide for PROMIS scores. *Journal of Patient-Reported Outcomes*, *4*(1), 1–7. 10.1186/s41687-020-0181-7 10.1186/s41687-020-0181-7 PMC7048882 32112189

[43] Jacobs P, Anello D, Elkin-Frankston S (2018). Using design to connect patients, providers, and researchers: a cognitive assessment and monitoring platform for integrative research (CAMPFIRE). International conference on applied human factors and ergonomics.

[44] Gold, H. T., Karia, R. J., Link, A., Lebwohl, R., Zuckerman, J. D., Errico, T. J., … Cantor, M. N. (2020). Implementation and early adaptation of patient-reported outcome measures into an electronic health record: a technical report. *Health Informatics Journal*, *26*(1), 129–140. 10.1177/1460458218813710. 10.1177/1460458218813710 30516095

[45] Braun V, Clarke V (2006). Using thematic analysis in psychology. Qualitative Research in Psychology.

[46] Masataka O, Kei I (2008). Color universal design (CUD)–how to make figures and presentations that are friendly to colorblind people.

[47] Hanmer, J., Jensen, R. E., & Rothrock, N. (2020). A reporting checklist for healthmeasures’ patient-reported outcomes: ASCQ-Me, Neuro-QoL, NIH toolbox, and PROMIS. *J Patient Rep Outcomes*, *4*(1), 1–7. 10.1186/s41687-020-0176-4 10.1186/s41687-020-0176-4 PMC7096598 32215788

